# Evaluation of Knowledge, Attitudes, and Skills in Evidence-Based Nursing Practice Among Master’s Degree Nursing Students

**DOI:** 10.3390/nursrep15040117

**Published:** 2025-03-25

**Authors:** Barbara Abram, Oliwia Radzimska, Jagoda Janiszewska, Aleksandra Świniarska, Roksana Papierkowska, Michał Czapla, Izabella Uchmanowicz

**Affiliations:** 1 Student Research Group for the Development of Nursing, Wroclaw Medical University, 50-367 Wroclaw, Polandjagoda.janiszewska@student.umw.edu.pl (J.J.); aleksandra.swiniarska@student.umw.edu.pl (A.Ś.); 2 Department of Nursing, Faculty of Nursing and Midwifery, Wroclaw Medical University, 51-618 Wroclaw, Poland; oliwia.radzimska@umw.edu.pl (O.R.); roksana.papierkowska@umw.edu.pl (R.P.); 3 Department of Emergency Medical Service, Faculty of Nursing and Midwifery, Wroclaw Medical University, 51-618 Wroclaw, Poland; michal.czapla@umw.edu.pl; 4 Group of Research in Care (GRUPAC), Faculty of Health Sciences, University of La Rioja, 26006 Logroño, Spain; 5 Centre for Cardiovascular Health, Edinburgh Napier University, Sighthill Campus, Edinburgh EH11 4DN, UK

**Keywords:** attitude, evidence-based nursing practice, knowledge, master’s degree, nursing students, skills

## Abstract

**Background**: Evidence-Based Nursing Practice (EBNP) plays a crucial role in ensuring high-quality patient care. This study evaluates master’s degree nursing students’ knowledge, attitudes, and skills related to EBNP, identifying strengths and key gaps that require curriculum improvements to enhance their competencies in evidence-based practice. **Methods**: A cross-sectional study was conducted among 103 master’s degree nursing students at Wrocław Medical University. Data were collected using a demographic questionnaire and the standardized Polish version of the Evidence-Based Practice Profile Questionnaire (EBP2Q). **Results**: The findings indicate that students demonstrated generally positive attitudes toward EBNP (mean score: 53.43 ± 10.05 out of 70). However, knowledge of research terminology was moderate (44.66 ± 18.01 out of 85), and the frequency of EBNP utilization in practice was relatively low (22.15 ± 8.74 out of 45). Significant differences were observed based on study mode and academic progression, with part-time students scoring higher in attitudes toward competency development (*p* = 0.02). A weak but positive correlation was found between professional experience and the frequency of EBNP utilization (r = 0.182, *p* = 0.068), while knowledge of research terminology showed a non-significant association with age (r = 0.167, *p* = 0.092). **Conclusions**: These findings highlight the need for targeted curriculum enhancements, particularly in research literacy, practical application opportunities, and the integration of mentorship and educational resources. Strengthening EBNP education will better equip nursing students to implement evidence-based practices in clinical settings, ultimately improving patient care quality.

## 1. Introduction

Evidence-Based Nursing Practice (EBNP) is a clinical nursing approach that integrates the best available scientific evidence, clinical expertise, and patient preferences to enhance patient care quality [1]. Originating from the broader concept of Evidence-Based Medicine (EBM), EBNP aims to ensure that nursing practices are grounded in research, systematically evaluated, and clinically relevant [2]. This shift towards evidence-based methodologies not only aims to improve care standards but also empowers nurses by providing them with decision-making tools based on rigorous scientific evidence [3,4]. EBNP is recognized as a key measure of healthcare quality. However, the literature indicates that nurses worldwide do not implement EBNP to the extent recommended by government agencies [5]. Key factors for successful implementation include identifying barriers and facilitators, as well as recognizing differences among nurses. Those with greater professional experience tend to demonstrate better skills and more positive attitudes toward the development of evidence-based practice, highlighting the need for curriculum changes in nursing education [6].

Nursing education plays a critical role in developing students’ competence in EBNP. Nursing academics have a special responsibility not only to impart knowledge but also to promote and support the implementation of EBNP [7,8]. This process includes teaching fundamental principles of EBP, such as effective evidence retrieval, formulating questions using the PICOT format (Population, Intervention, Comparison, Outcome, Timeframe), critically evaluating the information gathered, and integrating it into clinical practice 2. In doing so, nursing education equips students to apply EBNP in their daily professional work, bridging theory with practical application [9].

The foundational principle of Evidence-Based Nursing Practice (EBNP) is that nurses should be able to pose clinically relevant questions, understand research evidence, and apply findings in real-world settings. This requires a combination of skills, including familiarity with research terminology, critical thinking, and the ability to assess and implement evidence-based interventions [10,11]. However, despite its recognized benefits, fully integrating EBNP into clinical nursing practice remains challenging [12]. Factors such as variability in research literacy, limited access to resources, and organizational barriers can hinder the effective application of evidence-based practices in nursing [13].

Integrating Evidence-Based Nursing Practice (EBNP) not only improves the quality of patient care but also reduces complications, minimizes healthcare costs, and fosters professional responsibility among future nurses [14]. Studies have highlighted that EBNP adoption in Polish nursing faces challenges, with gaps in knowledge and practical application of evidence-based methods noted among practitioners [15]. A key issue in the nursing curriculum is the constraint of limited time and the lack of comprehensive evidence-based teaching, which hinders the adoption of EBNP. Despite the critical importance of EBNP, many nursing students demonstrate limited engagement and a significant knowledge gap. While some universities emphasize the significance of EBNP, they often fail to allocate sufficient resources for its integration into nursing curricula. According to Patelarou et al., this may be attributed to the high professional demands placed on students and the lack of necessary skills and knowledge [16]. Improving educational strategies to bridge these gaps is essential for producing nursing graduates who are competent in EBNP and capable of improving patient outcomes through scientifically informed practices [3,4,17,18,19]. Integrating EBNP into nursing not only increases the quality of patient care but also reduces complications, minimizes healthcare costs, and promotes professional responsibility among future nurses [14]. It enhances skill development and strengthens clinical expertise by incorporating research findings into daily practice. Despite its benefits, teaching and applying EBNP remains a global challenge, especially among nursing students early in their careers [20]. EBP is essential for ensuring patient safety, making it critical to equip nursing students with knowledge about Evidence-Based Nursing Practice. These challenges can be addressed by improving information management skills, such as searching for relevant studies in databases, critically appraising the information, and applying it effectively in clinical contexts [19].

The use of EBNP by unit nurses is crucial for improving healthcare quality and operational efficiency in clinical settings. As leaders of nursing teams, these nurses play a key role in implementing evidence-based practices in patient care [21]. Their proactive use of current scientific research and promotion of critical thinking in clinical decision-making not only reduces medical errors but also contributes to improved patient outcomes and safety [22]. Research indicates that nurses with more professional experience and access to resources like scientific databases and training programs are more likely to apply EBNP in clinical practice. However, ward nurses also serve as educators and mentors for junior team members, helping them to develop skills to find, critically analyze, and implement research findings [7]. For unit nurses to fully realize the potential of EBNP, they must have access to specialized training, adequate time to enhance their competence in evidence analysis, and strong institutional support. Implementing these measures will enable nurses to promote evidence-based practice more effectively and further elevate the standards of nursing care [9]. Additionally, monitoring the effects of implemented EBNP solutions is essential for assessing their impact on both healthcare professionals and patient outcomes [21]. A higher level of readiness to adopt EBNP among nurses, particularly those pursuing master’s degrees, is associated with improved clinical decision-making and enhanced patient care [23].

The implementation of EBNP is crucial for improving healthcare quality and professional competence. However, despite its well-documented benefits, the application of EBNP in nursing faces significant barriers, including inadequate education and limited access to resources [24]. These challenges are particularly evident among nursing students, for whom integrating EBNP requires strengthening evidence-based practice components within educational programs.

The primary aim of this study is to evaluate master’s degree nursing students’ knowledge, attitudes, and skills in Evidence-Based Nursing Practice (EBNP). Specifically, it examines their familiarity with EBNP terminology, attitudes toward EBNP, competency in applying its principles, and the frequency of its use in clinical settings. The study also investigates how age and seniority may influence these competencies. The findings aim to identify strengths and areas for improvement in nursing education, ultimately guiding curriculum enhancements to better integrate EBNP into future nursing practice.

## 2. Materials and Methods

### 2.1. Study Design and Settings

This study was cross-sectional and conducted at the Medical University in Wrocław. Its objective was to assess the knowledge, attitudes, and skills of nursing students regarding Evidence-Based Nursing Practice.

This study included 103 master’s degree nursing students. Most participants were female (95.41%). The questionnaire was distributed online via the university’s student platform, ensuring equal access for all participants. A total of 103 students accessed the questionnaire, and all 103 completed it, resulting in a 100% response rate. Participants were informed about the purpose of this study, as well as the principles of anonymity and voluntary participation. Each student had the right to withdraw from this study at any stage.

### 2.2. Research Instruments

This study utilized two research instruments. A custom questionnaire included demographic questions (age, gender, place of residence, study mode, year of study, professional experience), as well as questions assessing general knowledge and the application of EBNP principles. The second instrument was the standardized Polish version of the original English Evidence-Based Practice Profile Questionnaire (EBP2Q) [1,25]—a validated tool designed to evaluate six key aspects of EBP, with high internal reliability (Cronbach’s alpha ranging from 0.783 to 0.959):

- Relevance—Attitudes toward developing EBP competencies;
- Sympathy—General attitudes toward EBP;
- Terminology—Knowledge of research terminology;
- Practice—Frequency of EBP application in practice;
- Confidence—Skills related to EBP;
- Non-Domain Items—Additional aspects of EBP.

The questionnaire consists of 74 items, measured using a Likert-type scale, where responses range from 1 (strongly disagree) to 5 (strongly agree), depending on the domain. The total scores for each domain vary due to differences in the number of items, making direct comparisons across domains methodologically inappropriate. Instead, higher scores within each domain indicate greater proficiency, confidence, or engagement in that specific aspect of EBP. The Polish version of EBP2Q underwent a rigorous validation process, confirming its psychometric robustness and applicability in assessing EBP-related knowledge, attitudes, and skills among Polish nursing students [25].

### 2.3. Statistical Analysis

Quantitative variable analysis (i.e., numerical data) was conducted by calculating the mean, standard deviation, median, quartiles, minimum, and maximum. Qualitative variable analysis (i.e., non-numerical data) was performed by determining the frequency and percentage of each value. Comparison of quantitative variables between two groups was carried out using the Mann–Whitney test. Correlations between quantitative variables were analyzed using Spearman’s correlation coefficient. A significance level of 0.05 was adopted for the analysis. Consequently, all *p*-values below 0.05 were interpreted as indicating statistically significant associations. The analysis was performed using R software, version 3.6.2.

## 3. Results

The majority of participants were women (95.15%), men accounted for 3.88%, and missing data accounted for 0.97%. The mean age of the participants was 27.46 years (SD = 7.83), and the vast majority (78.64%) lived in urban areas, while 21.36% lived in rural areas. The majority of respondents were part-time students (61.17%) compared to 38.83% who were full-time students. Participants were divided into two cohorts, with second-year students (66.99%) outnumbering first-year students (33.01%). A significant proportion of respondents had work experience (88.35%), with a mean work experience of 52.84 months (SD = 95.41). Table 1 illustrates the demographic characteristics of the study group.

The findings reveal generally positive attitudes and moderate familiarity with EBNP among students. The mean score for competency development attitudes was 53.43 out of 70, while knowledge of research terminology showed variability, with a mean of 44.66 out of 85. The frequency of EBNP utilization was moderate, indicating room for improvement in consistent EBNP application. Statistically significant differences were observed based on study mode and year, with part-time students showing stronger competency development attitudes.

Table 2 shows the results of the EBP2 questionnaire, which assesses knowledge, attitudes, and skills related to Evidence-Based Nursing Practice. For “Attitudes toward competence development in EBNP”, the mean score was 53.43 out of a possible 70 points, indicating that students have a positive attitude toward improving their skills in this area. For “Attitudes toward EBNP”, the mean score was 22.4 out of 35 points, reflecting a generally favorable view of Evidence-Based Nursing Practice. Knowledge of research terminology was more mixed, with a mean score of 44.66 out of 85, suggesting moderate familiarity with the field. The frequency of use of EBNP in practice was rated at a mean of 22.15 out of 45 points, indicating insufficient implementation of these methods. In the “EBNP-related skills” category, students scored a mean of 41.08 out of 55 points, while in the “Other aspects of EBNP” category, the mean was 54.03 out of 80 points, revealing significant differences between the level of theoretical and practical knowledge.

For attitudes toward competency development and general attitudes toward EBNP, no significant correlations with age or experience were observed, indicating stable attitudes regardless of these factors. Similarly, skills related to EBNP showed no meaningful association with age or tenure.

A slight positive trend was noted for knowledge of research terminology with age (r = 0.167, *p* = 0.092) and frequency of EBNP utilization with professional experience (r = 0.182, *p* = 0.068), though neither was statistically significant. Other aspects of EBNP, such as critical thinking, exhibited a near-significant correlation with professional experience (r = 0.191, *p* = 0.056) (Table 3).

Analysis of the data presented in Table 4 indicates some differences in the EBP2 questionnaire results, which can be attributed to the mode of study and year of study. Part-time students, who often have more work experience, scored higher in attitudes toward developing EBNP competencies, suggesting a positive impact of work experience on their attitudes toward this methodology. First-year students, on the other hand, showed higher scores in the attitudes toward EBNP, which may indicate greater enthusiasm or expectations for beginning their studies. However, no significant differences were found in knowledge of research terminology or EBNP-related skills, highlighting the need for greater emphasis on developing these areas in educational programs regardless of demographics. In particular, it is worth focusing on the practical aspects of education and providing access to resources that can help students to effectively implement knowledge in clinical settings.

## 4. Discussion

The results of this study indicate both strengths and areas for improvement in nursing students’ knowledge, attitudes, and skills regarding Evidence-Based Nursing Practice (EBNP). While nursing students generally demonstrate positive attitudes toward EBNP, as reflected in the scores for competency development, there are significant gaps in their knowledge and practical application. These findings suggest that students recognize the importance of evidence-based practices in enhancing patient care, aligning with broader healthcare trends, where evidence-based approaches are increasingly emphasized to ensure high-quality, efficient, and safe care [3,4]. According to Y. Xia et al., EBNP is crucial in addressing nursing challenges, improving care quality, and enhancing clinical outcomes. By minimizing the gap between scientific evidence and practice, EBNP helps the nursing profession to progress toward a more evidence-based and professional framework. The current study, using the EBP2 questionnaire, reveals moderate familiarity with research terminology and low frequency of EBNP application in practice. These findings suggest that the potential benefits of EBNP, such as improved clinical outcomes and bridging the evidence-practice gap, are not being fully realized [1].

Wakibi argues that educational strategies for Evidence-Based Nursing Practice (EBNP) should be multidimensional, be student-centered, and bridge theory with practice. Key strategies include simulated clinical scenarios that allow students to apply evidence-based data in decision-making and teaching the connections between EBNP elements to enhance decision-making [20]. Problem-based learning (PBL) encourages critical thinking and the application of EBNP principles by solving clinical problems. This study highlights the need to strengthen practical components in nursing education, such as information retrieval, evidence analysis, and mentorship from experienced nurses. Both sources emphasize the importance of tailoring methods to student needs and contextual factors, such as limited resources in different countries [20].

The variability in students’ knowledge of research terminology highlights a key area for improvement in EBNP. Understanding and interpreting research findings requires familiarity with research language and methodology. A comparison with Belowska et al.’s results, where 30% of students had never encountered the term EBNP and 40% had never assessed research quality, underscores the need for stronger education in this area. In this study, the students’ mean score for knowledge of research terminology was 44.66 out of 85, with moderate use of EBNP in practice (22.15 out of 45). These results indicate that EBNP knowledge should be enhanced through modules focused on the research methodology, critical evaluation, and practical application of research findings [26].

The moderate scores for EBNP utilization further highlight the gap between theoretical knowledge and practical application. Although students show positive attitudes toward EBNP, applying these concepts in clinical practice requires structured opportunities and encouragement within educational settings. The challenges in implementing EBNP may stem from the attitudes of nurses, including educators. According to Młynarska et al., older nurses had lower knowledge and skills related to EBNP and applied it less frequently in practice. Professional experience was found to influence EBNP use, with more experience correlating to less frequent application of evidence-based practices [5]. In comparison, students with professional experience showed a better attitude toward developing EBNP competencies than practicing nurses.

Significant differences based on study mode and year suggest that factors such as clinical exposure and academic progression influence EBNP competencies. Part-time students, likely to have more professional experience, scored higher in attitudes toward competency development, indicating that practical experience may reinforce the value of EBNP. Similarly, second-year students scored higher in other EBNP aspects, reflecting the impact of academic progression on their understanding of EBNP principles. These findings highlight the importance of experience and curriculum structure in developing EBNP competencies. Tailoring educational approaches to different levels of experience and academic progress could further support the development of evidence-based skills. Insights from Halili X et al. emphasize that partnerships between academic institutions and clinical practice are crucial in overcoming obstacles to EBNP adoption [1]. The American Association of Colleges of Nursing (AACN) supports such collaborations, as they enhance nurses’ engagement in clinical settings and equip them to lead transformative changes in healthcare [27,28].

The findings align with broader challenges in implementing EBNP in Polish nursing contexts, where structural and organizational barriers limit the effective application of evidence-based methods. Organizational support, mentorship, and resources are essential for fostering an environment where nursing students can develop and apply EBNP skills. Integrating mentorship from experienced practitioners, providing access to research databases, and creating a supportive clinical culture could significantly enhance students’ engagement with EBNP.

To bridge the identified gaps, nursing curricula should emphasize research literacy and the practical application of EBNP. Research by Li et al. highlights the significant impact of educational programs on EBNP competencies, critical thinking, and decision-making abilities. Li stresses the importance of tailoring programs to focus on the practical aspects of EBNP, particularly critical appraisal of evidence. The study shows that strong evidence-based practices enhance the effectiveness of student education [27]. Additionally, incorporating case-based learning and simulations that mirror clinical decision-making can help students to transition from theoretical understanding to effective practice.

Majers and Warshawsky discuss the critical role of nurse leaders in implementing Evidence-Based Management (EBM) [21]. They emphasize that nurse leaders, using evidence-based practices, can effectively address challenges such as the dynamic changes in the healthcare system, including the COVID-19 pandemic. EBM helps to bridge the gap between scientific evidence and practice, improving management efficiency and healthcare outcomes [21].

According to Berends E., the use of Evidence-Based Management (EBM) bridges the gap between scientific findings and their application in decision-making. By integrating scientific evidence into management, leaders can directly apply the best available data to improve strategies, eliminating barriers caused by the insufficient transfer of knowledge from the literature to practice. EBM helps to implement evidence-based standards in organizational settings, reducing reliance on intuition and limited data and improving both organizational performance and service quality [29].

A significant correlation emerges regarding age, with younger individuals showing greater receptiveness to EBNP. In this study, younger students demonstrated high engagement in EBNP activities, including regularly reviewing the literature and sharing research outcomes. Furthermore, 82.4% expressed positive attitudes toward EBNP, recognizing its value in their work. However, 46.8% rated their skills in locating and evaluating evidence as excellent, and 61.9% assessed their ability to formulate research questions highly. These findings highlight the need for targeted training to enhance skills like research question formulation and proficient use of evidence-based tools.

Nonetheless, certain areas require further development. Only 46.8% of respondents rated their skills in locating and evaluating evidence as excellent, while 61.9% assessed their ability to formulate research questions highly. These findings highlight the need for targeted training to enhance competencies such as research question formulation and the proficient use of evidence-based tools.

Information-seeking behaviors of nurses and nursing students have long been a focus of research. Numerous studies emphasize individual and organizational factors that affect information-seeking activities. Key determinants of success include learning styles, confidence levels, perceptions of EBNP, search skills, available time, and access to resources and technology. Notably, confidence has been found to significantly influence nursing students’ use of knowledge sources in the first two years of professional practice after graduation [30,31,32]. To advance nursing science and improve patient outcomes, it is crucial to educate nursing students on not only the value of evidence-based knowledge but also how to access, critically evaluate, and apply it in clinical situations when necessary [14].

### 4.1. Study Limitations

First, this study has several limitations that should be considered when interpreting the results. The sample consisted of 103 nursing students from a single university, which may limit the generalizability of the findings to the broader population of nursing students. Future research should include a more diverse sample from multiple institutions to provide a broader perspective on EBNP competencies. Second, this study relied on self-reported questionnaires, which can introduce response bias. Participants may have overestimated or underestimated their knowledge and skills, particularly due to social desirability bias. Future studies could benefit from complementary objective assessments, such as practical skill evaluations or observed clinical decision-making. Lastly, the cross-sectional design provides a snapshot of students’ knowledge, attitudes, and skills at a single point in time and does not capture changes over time or the long-term impact of EBNP education. A longitudinal study following students throughout their academic and professional journey could provide deeper insights into how EBNP competencies develop. Additionally, a key limitation of this study is the lack of detailed information on students’ previous undergraduate education in EBNP. Since the participants were master’s students who had completed their bachelor’s degrees at various institutions, their prior exposure to EBNP training may have varied significantly. This heterogeneity in educational backgrounds could influence their competencies and perceptions of EBNP, introducing an additional confounding factor. Future research should consider collecting more detailed data on students’ previous EBNP training to better contextualize the findings. Despite these limitations, this study offers valuable insights into the current state of EBNP education, highlighting areas for improvement in nursing curricula and the need for stronger integration of evidence-based practice in clinical training.

### 4.2. Practical Implications

The findings of this study emphasize the need to enhance EBNP integration into nursing education by improving research literacy and practical application. Blended learning approaches, combining lectures with case-based discussions and simulations, can help students to develop both theoretical knowledge and practical skills. Interdisciplinary learning with medical and allied health students can further improve teamwork and evidence-based decision-making. A major barrier to EBNP adoption is limited faculty training in evidence-based teaching. Universities should provide faculty development programs and integrate EBNP content across multiple courses rather than limiting it to standalone modules. Expanding access to scientific databases and offering structured research training will enhance students’ ability to engage with evidence. Additionally, mentorship programs pairing students with experienced clinicians or faculty researchers could strengthen their confidence in applying EBNP. Implementing these strategies will better equip nursing students for evidence-based clinical practice, ultimately improving patient outcomes and advancing the quality of nursing care.

## 5. Conclusions

The findings of this study highlight the need for improvements in nursing education, particularly in research literacy and the practical application of Evidence-Based Nursing Practice (EBNP). While students demonstrated positive attitudes toward EBNP, gaps were observed in their knowledge of research terminology and the frequency of EBNP utilization in practice. These findings suggest that nursing curricula should place greater emphasis on integrating research methodology, critical appraisal skills, and hands-on application of EBNP principles.

To enhance EBNP integration, nursing programs should incorporate structured training in literature searching, appraisal of scientific evidence, and practical workshops focused on applying EBNP concepts in clinical decision-making. Additionally, active learning strategies, such as problem-based learning and simulation-based training, could help to bridge the gap between theoretical knowledge and real-world application. Expanding access to scientific databases and evidence-based practice toolkits would further equip students with the necessary resources to develop research literacy and critical thinking. Collaboration between academic institutions and clinical settings should also be strengthened to facilitate mentorship programs where students can engage with experienced nurses applying EBNP in daily practice.

Addressing these areas through enhanced educational strategies, mentorship programs, and increased clinical exposure could facilitate the broader adoption of EBNP, ultimately improving patient care quality and nursing practice standards. Future research should explore longitudinal approaches to assess how EBNP competencies develop over time and identify the most effective educational interventions for fostering evidence-based decision-making among nursing students.

## Figures and Tables

**Table 1:** Master’s degree nursing students’ characteristics.

Characteristic	Value	N—Mean	SD
Gender	Women	98	95.15%
Gender	Men	4	3.88%
Gender	No data	1	0.97%
Age	[years]	27.46	7.83
Place of Residence	City	81	78.64%
Place of Residence	Village	22	21.36%
Study Mode	Full-time	40	38.83%
Study Mode	Part-time	63	61.17%
Year of Study	First Year	34	33.01%
Year of Study	Second Year	69	66.99%
Professional Experience	Yes	91	88.35%
Professional Experience	No	12	11.65%
Seniority	[months]	52.84	95.41

**Table 2:** EBP2 results.

EBP2	Score Range	N	Mean	Standard Deviation	Median	Min	Max	Quartile 1	Quartile 3	Narrative Results
Attitude Toward Competency Development in EBNP	14–70	103	53.43	10.05	56	14	70	48	60	Positive attitudes toward competency development, though some variability exists.
Attitude Toward EBNP	7–35	103	22.4	4.02	22	11	33	20	25	Generally favorable views, but with some differences across individuals.
Knowledge of Research Terminology	17–85	103	44.66	18.01	46	17	85	28.5	60.78	Moderate knowledge of terminology, highlighting a need for further education.
Frequency of EBNP Utilization	9–45	103	22.15	8.74	21	9	45	16	28	Moderate frequency of EBNP utilization, indicating room for more consistent application.
Skills Related to EBNP	11–55	103	41.08	6.8	43	19	55	38	44	Moderate competency in applying EBNP skills, but more practice is needed.
Other Aspects of EBNP	16–80	103	54.03	7.25	53	38	80	49	57.8	Strong theoretical understanding, but practical application needs enhancement.

**Table 3:** Correlations between EBP2 scores and demographic variables (age and work experience).

EBP2	Age	Seniority
Attitude Toward Competency Development in EBNP	r = −0.043, *p* = 0.668	r = −0.028, *p* = 0.781
Attitude Toward EBNP	r = −0.16, *p* = 0.107	r = −0.153, *p* = 0.126
Knowledge of Research Terminology	r = 0.167, *p* = 0.092	r = 0.126, *p* = 0.209
Frequency of EBNP Utilization	r = 0.133, *p* = 0.181	r = 0.182, *p* = 0.068
Skills Related to EBNP	r = −0.104, *p* = 0.296	r = −0.011, *p* = 0.915
Other Aspects of EBNP	r = 0.141, *p* = 0.156	r = 0.191, *p* = 0.056

**Table 4:** Comparison of EBP2 scores by gender, place of residence, study mode, year of study, and professional experience.

Domain	EBP2	Gender	Gender	*p*	Place of Residence	Place of Residence	*p*	Study Mode	Study Mode	*p*	Year of Study	Year of Study	*p*	Professional Experience	Professional Experience	*p*
		Woman (N = 98)	Men (N = 4)		City (N = 81)	Village (N = 22)		Full-Time Students (N = 40)	Part-Time Students (N = 63)		I Year (N = 34)	II Year (N = 69)		Yes (N = 91)	No (N = 12)	
Attitude Toward Competency Development in EBNP	mean ± SD	53.33 ± 10.2	56.5 ± 7.42	0.756	52.95 ± 10.45	55.18 ± 8.42	0.42	51.31 ± 9.09	54.77 ± 10.46	0.02	53.47 ± 11.72	53.4 ± 9.21	0.516	53.46 ± 10.09	53.17 ± 10.15	0.865
Attitude Toward Competency Development in EBNP	median	56	54.5		54	56		52.5	56		56	54		56	54.5	
Attitude Toward Competency Development in EBNP	quartiles	48–60	52.25–58.75		47.38–60	52.25–59.5		45–56	51–61.5		50.25–60	48–59		49–59.5	44.5–61.25	
Attitude Toward EBNP	mean ± SD	22.49 ± 4.01	21.75 ± 4.11	0.671	22.53 ± 3.99	21.91 ± 4.19	0.396	21.68 ± 3.81	22.86 ± 4.11	0.075	23.71 ± 4.39	21.75 ± 3.69	0.012	22.46 ± 4.08	21.92 ± 3.7	0.496
Attitude Toward EBNP	median	22	21		22	22		21	23		24	22		22	21	
Attitude Toward EBNP	quartiles	20–25	18.75–24		20–25	18.25–24.75		19.75–23.25	20–25.5		20.25–26.75	20–24		20–25	20.75–22.25	
Knowledge of Research Terminology	mean ± SD	44.11 ± 18.03	53.5 ± 16.66	0.224	43.79 ± 17.96	47.88 ± 18.28	0.338	41.72 ± 16.68	46.54 ± 18.7	0.202	42.43 ± 17.61	45.77 ± 18.23	0.367	44.76 ± 17.65	43.97 ± 21.45	0.762
Knowledge of Research Terminology	median	44.6	57.5		44	50.5		39	47		44.1	47		46	43.5	
Knowledge of Research Terminology	quartiles	27.25–59.5	46–65		28–58	33.25–63		29.75–51.5	27.5–63		26.5–50.75	30–62		28.5–60.78	30.5–54	
Frequency of EBNP Utilization	mean ± SD	22.12 ± 8.82	21.5 ± 8.43	0.809	22.11 ± 8.89	22.3 ± 8.36	0.747	21.65 ± 8.59	22.46 ± 8.89	0.551	20.53 ± 7.72	22.94 ± 9.15	0.245	22.42 ± 8.43	20.04 ± 11.03	0.257
Frequency of EBNP Utilization	median	21	21		19	23		20	21		18	23		23	17	
Frequency of EBNP Utilization	quartiles	16–28	15.25–27.25		16–28	17.5–27.5		15–27	17–29.5		17–26	16–29		16.5–28	12–27.5	
Skills Related to EBNP	mean ± SD	41.12 ± 6.78	37.25 ± 4.92	0.113	40.72 ± 7.05	42.41 ± 5.7	0.277	41.27 ± 6.08	40.96 ± 7.26	0.709	40.15 ± 7.86	41.54 ± 6.22	0.975	41.13 ± 6.86	40.67 ± 6.61	0.502
Skills Related to EBNP	median	43	37		42	43.5		42	43		43	42		43	42	
Skills Related to EBNP	quartiles	38.25–44	33–41.25		38–44	39.25–45		37.75–44	38.5–44.5		37.5–44	38–44		39–44.5	35.75–43.25	
Other Aspects of EBNP	mean ± SD	53.75 ± 6.83	54.5 ± 7.05	0.749	53.97 ± 7.64	54.28 ± 5.73	0.678	54.01 ± 7.44	54.05 ± 7.18	0.799	51.62 ± 6.11	55.23 ± 7.5	0.035	54.25 ± 7.48	52.38 ± 5.07	0.291
Other Aspects of EBNP	median	52.5	54.5		54	52.5		52.5	54		51.5	54		54	50	
Other Aspects of EBNP	quartiles	49–57.45	51.25–57.75		49–58	51–57.33		49–56.9	50–57.8		48–55.6	50–60		50–58	49–53.13	

## Data Availability

The raw data supporting the conclusions of this article will be made available by the authors on request.

## Public Involvement Statement

There was no public involvement in any aspect of this research.

## Guidelines and Standards Statement

This manuscript was drafted in accordance with the Strengthening the Reporting of Observational Studies in Epidemiology (STROBE) guidelines for cross-sectional studies [34].

## Abbreviations

The following abbreviations are used in this manuscript:

EBNP	Evidence-Based Nursing Practice
EBM	Evidence-Based Medicine
EBP2Q	Evidence-Based Practice Profile Questionnaire
